# Prevalence of chronic kidney disease and associated factors among the Chinese population in Taian, China

**DOI:** 10.1186/1471-2369-15-205

**Published:** 2014-12-21

**Authors:** Lingyu Xue, Yanxia Lou, Xiaoqing Feng, Chuaihai Wang, Zhangshen Ran, Xuebin Zhang

**Affiliations:** Department of Nephrology, the Affiliated Hospital of Taishan Medical University, No. 706 Taishan Street, Taian, 271000 Shandong Province China; Department of Nephrology, Veterans Hospital, Taian, 271000 Shandong Province China; The Physical Examination Centre of Affiliated Hospital of Taishan Medical University, Taian, 271000 Shandong Province China

**Keywords:** Chronic kidney disease, Epidemiology, Prevalence, Risk factors, Awareness

## Abstract

**Background:**

This study was designed to assess the prevalence of chronic kidney disease (CKD) and associated risk factors among the Chinese population in Taian, China.

**Methods:**

A primary care-based cross-sectional study was conducted in Taian, China, from September to December 2012. Participants selected by a multi-stage stratified cluster sampling procedure were interviewed and tested for hematuria, albuminuria, estimated glomerular filtration rate (eGFR) and other clinical indices. Factors associated with CKD were analyzed by univariate and multivariate logistic regression analysis.

**Results:**

A total of 14,399 subjects were enrolled in this study. The rates of hematuria, albuminuria and reduced eGFR were 4.20%, 5.25% and 1.89%, respectively. Approximately 9.49% (95% CI: 8.93%–10.85%) of the participants had at least one indicator of CKD, with an awareness of 1.4%. Univariate analyses showed that greater age, body mass index, and systolic and diastolic blood pressure; higher levels of serum creatinine, uric acid, fasting blood glucose, triglycerides, total cholesterol and low-density lipoprotein cholesterol; and lower eGFR were associated with CKD (*p* < 0.05 each). Multivariate analysis showed that age, female gender, educational level, smoking habits, systolic blood pressure, and history of diabetes mellitus, hyperlipidemia, hypercholesterolemia and hyperuricemia were independent risk factors for CKD.

**Conclusions:**

The prevalence of CKD in the primary care population of Taian, China, is high, although awareness is quite low. Health education and policies to prevent CKD are urgently needed among this population.

## Background

Chronic kidney disease (CKD), as defined and classified by the Kidney Disease Outcomes Quality Initiative (K/DOQI) of the National Kidney Foundation (NFK) [1], is one of the most important chronic diseases worldwide [2, 3]. Up to 14% of adults in the United States aged > 18 years, representing an estimated 31.4 million people, were found to have some degree of CKD in 2007–2010 [4]. In Australia, CKD was common present in approximately 1 in 7 persons aged ≥ 25 years [5]. In addition to being common in developed nations, CKD is also highly prevalent in developing countries [6]. A cross-sectional study indicated that the nationwide prevalence of CKD in China was 10.8% (95% confidence interval [CI], 10.2–11.3), affecting an estimated 119.5 million (95% CI, 112.9–125.0 million) patients, similar to the level observed in the United States in 2003 [7, 8]. For the major outcomes of CKD are progression to kidney failure, end-stage renal disease (ESRD), complications of decreased kidney function, and cardiovascular disease, CKD can greatly affect the general population, especially individuals at high-risk for hypertension or diabetes [9–13]. Furthermore, the rapid increase in the prevalence of risk factors, such as diabetes, hypertension, and obesity, has increased the burden of CKD, making CKD an important socioeconomic and public health problem [6, 14].

CKD is considered to be a multi-factorial disease, with genetic and environmental factors contributing to its pathogenesis [15]. Many factors are associated with the prevalence of CKD including gender, occupation, education, marital status, diabetes, hypertension, hyperuricemia, history of kidney stones, and the use of traditional medicines [16, 17]. To better prevent and control this disease, several studies in China have investigated the characteristics and potential risk factors of CKD [18–20]. However, little is known about the epidemiology of CKD in Taian, China. This study was therefore designed to evaluate the epidemiology of CKD among a primary care population in Taian, China, from September to December 2012.

## Methods

### Sampling and subjects

This cross-sectional survey was conducted between September and December 2012. A multi-stage stratified cluster sampling procedure was employed to select a representative sample of the primary care population in Taian, China. Briefly, two tertiary hospitals (Affiliated Hospital of Taishan University and Taian Central Hospital) and two secondary hospitals (Second Chinese Medicine Hospital in Taian and Taian Rongjun Hospital) were randomly chosen based on the population distribution in Taian. Eighty primary care units from each hospital, or a total of 320 units, were randomly selected; half of the units were located in the Taishan District and half in the Daiyue District. Subsequently, 50 subjects, aged ≥ 18 years and living in Taian > 6 months, were randomly enrolled from each of the 320 units. All participants provided written informed consent, and the study protocol was approved by the Ethics Committee of Affiliated Hospital of Taishan University.

### Questionnaire

A structured questionnaire was designed for data collection. Factors analyzed included sociodemographic status (name, age, gender, ethnicity, educational level, marital status, financial situation and contact information), health-related behaviors (smoking, alcohol drinking, oil intake, diet, spending on food and type of insurance), awareness of CKD and personal and family history of relevant diseases (hypertension, diabetes mellitus, chronic kidney disease, hyperlipidemia and other diseases). All participants were personally interviewed by well-trained interviewers using uniform and standardized language.

### Anthropometric measurements

Standard protocols and techniques were utilized by medical staff to measure anthropometric parameters, including weight, height, systolic blood pressure (SBP), and diastolic blood pressure (DBP). Body mass index (BMI) was calculated as weight (Kg)/height (m^2^).

### Blood and urine sample collection

Morning urine samples (non-menstrual period for women) were obtained after an overnight fast (at least 10 h). Urinary factors were measured using a Urine Analyzer (Urit Group, China) and urinary sediments were examined by light microscopy (Olympus Corporation, Japan). Venous blood samples were collected at the same time and used to measure various biomarkers, including fasting blood glucose (FBG), total cholesterol (TCH), triglyceride (TG), high density lipoprotein-cholesterol (HDL-C), low density lipoprotein-cholesterol (LDL-C), uric acid (UA) and serum creatinine (Scr) concentrations. All assays were performed by well-trained laboratory technicians using reagents from BioSino Bio-technology and Science Inc, China. Differences between males and females and between subjects with and without CKD were analyzed.

### Definitions of variables

CKD was defined as kidney damage, ascertained by the presence of albuminuria and/or hematuria, or estimated glomerular filtration rate (eGFR) < 60 mL/min/1.73 m^2^, irrespective of cause [21–23]. Albuminuria was defined as urinary albumin concentration > 20 mg/L, measured immunoturbidimetrically [7]. Subjects with hematuria of ≥ 1+ were confirmed by microscopic analysis, with three or more red blood cells in urinary sediments considered abnormal [21]. Scr was measured with Jaffe’s kinetic method and eGFR was calculated by the Modification of Diet in Renal Disease (MDRD), modified for Chinese subjects; i.e., eGFR (mL/min/1.73 m^2^) = 175 × (Scr) - 1.234 × (age) - 0.179 × (0.742 if female) [24].

Awareness of CKD was defined as subject knowledge of having CKD, based on a previous diagnosis by a physician. Hypertension was defined as SBP ≥ 140 mmHg and/or DBP ≥ 90 mmHg, any use of antihypertensive medication, or self-reported history of hypertension. Obesity was defined as BMI ≥ 28 kg/m^2^. Diabetes mellitus was defined as fasting blood glucose (FBG) ≥ 7.0 mmol/L and/or postprandial blood glucose (PBG) ≥ 11.1 mmol/L, or a history of diabetes. Hyperlipidemia (HLP) was defined as TG ≥ 1.70 mmol/L and/or TCG ≥ 5.72 mmol/L, or a history of HLP. Hyperuricemia was defined as UA ≥ 420 μmol/L for males and ≥ 360 μmol/L for females, or a history of hyperuricemia.

### Statistical analysis

All statistical analyses were performed using SPSS 15.0 software. Categorical variables were presented as percentages and compared using Pearson chi-square tests, and continuous variables were reported as mean ± standard deviation (SD) and compared using unpaired *t*-tests or one-way analysis of variance. Multivariate logistic regression was performed to identify independent factors associated with PKD, including age, gender, smoking, educational level, obesity, history of nephrotoxic medications and hypertension, diabetes, cardiovascular disease, hyperlipidemia, hyperuricemia, and other diseases. Odds ratios (ORs) and corresponding 95% CIs were calculated. A *p*-value < 0.05 was considered statistically significant.

## Results

### Participant characteristics

After excluding 1,601 subjects who lacked sufficient data, 14,399 (90.0%) participants were included in this analysis; of these, 59.46% were males and 40.54% were females. Baseline demographic, anthropometric and laboratory data are shown in Table 1. The mean age of all subjects was 48.97 ± 17.02 years (range, 18–89 years), while the mean ages of males and females were 49.64 ± 16.65 and 48.13 ± 18.01 years, respectively. Age, BMI, SBP, DBP, Scr, UA, FBG, TG, and eGFR were significantly higher in males (*p* < 0.05 each), whereas HDL-C and LDL-C were significantly higher in female (*p* < 0.05 each). TCH was similar in males and females (*p* > 0.05).

**Table 1:** **Sociodemographic and clinical characteristics of the participants**

Characteristics	Total ***(*** ***± s)***	Males ***(*** ***± s)***	Female ***(*** ***± s)***	***t/χ*** ^***2***^	***p-value***
Number	14 399	8 561	5 838	-	*-*
Age (years)	48.97 ± 17.02	49.64 ± 16.65	48.13 ± 18.01	3.52	*0.001*
BMI (kg/m^2^)	24.29 ± 3.12	25.58 ± 3.43	23.51 ± 3.52	31.09	*0.001*
SBP (mmHg)	124.32 ± 18.56	128.78 ± 18.24	120.72 ± 17.39	15.23	*0.000*
DBP (mmHg)	72.38 ± 11.93	85.12 ± 13.92	75.93 ± 11.57	27.12	*0.000*
Scr (μmol/L)	79.10 ± 29.18	90.32 ± 20.78	65.29 ± 14.71	50.23	*0.000*
UA (μmol/L)	286.28 ± 89.04	297.5 ± 90.60	271.31 ± 88.2	17.22	*0.000*
FBG (mmol/L)	5.8 ± 1.61	5.85 ± 1.66	5.73 ± 1.56	4.39	*0.000*
TG (mmol/L)	1.57 ± 1.16	1.62 ± 1.23	1.47 ± 1.14	7.63	*0.000*
TCH (mmol/L)	4.85 ± 1.05	4.84 ± 1.00	4.87 ± 1.07	-1.64	*0.101*
HDL-C (mmol/L)	1.46 ± 0.42	1.43 ± 0.40	1.53 ± 0.54	-13.05	*0.000*
LDL-C (mmol/L)	2.78 ± 0.80	2.77 ± 0.79	2.80 ± 0.81	-2.81	*0.005*
Hematuria (%)	605(4.20%)	197(2.30%)	408(6.9%)	120.72	*0.000*
Albuminuria (%)	756(5.25%)	533(6.23%)	223(3.82%)	49.78	*0.000*
eGFR (mL/min/1.73 m^2^)	101.23 ± 36.03	102.74 ± 35.30	100.85 ± 37.42	3.08	*0.002*

BMI, body mass index; SBP, systolic blood pressure; DBP, diastolic blood pressure; Scr, serum creatinine; UA, uric acid; FBG, fasting blood glucose; TG, triglyceride; TCH, total cholesterol; HDL-C, density lipoprotein-cholesterol; LDL-C, low density lipoprotein-cholesterol; eGFR, estimated glomerular filtration rate.

### Prevalence of CKD

**Figure 1:**
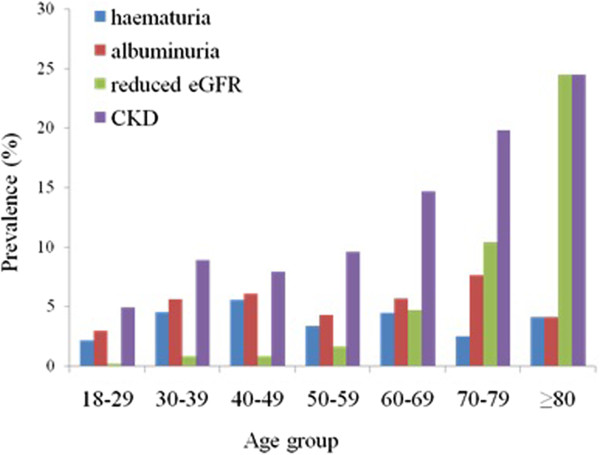
**Age-specific prevalence of indicators of chronic kidney disease.**

Of the 14,399 subjects, 1,366 (9.49%; 95% CI: 8.93%–10.85%), including 747 males and 619 females, were positive for CKD. The rates of hematuria, albuminuria and reduced eGFR (<60 mL/min/1.73 m^2^) in subjects with CKD were 4.20%, 5.25% and 1.89%, respectively, although only 1.4% of these subjects were aware they had CKD. The prevalence of CKD was similar in males and females, while the rates of albuminuria and reduced eGFR were significantly higher in males (*p* < 0.01 each) and the rate of hematuria was significantly higher in females (*p* = 0.002). Figure 1 shows the age-specific rates of CKD, hematuria, albuminuria and reduced eGFR. Rates of CKD, albuminuria and reduced eGFR differed significantly by age (*p* < 0.01 each), with the rats of CKD and reduced eGFR increasing with age, especially among people aged > 60 years. In contrast, the rate of albuminuria was highest in subjects aged 40–49 years. Reduced eGFR was observed in 24.49% of subjects aged > 80 years.

### CKD Risk factors

To characterize the risk factors for CKD, anthropometric measurements and biochemical indices were compared in subjects with and without CKD. Univariate analysis showed that age, BMI, SBP, DBP, Scr, UA, FBG, TG, TCH, and LDL-C were significantly higher, while eGFR was significantly lower, in subjects with than without CKD (*p* < 0.05 each) (Table 2).

**Table 2:** **Univariate analysis of factors in subjects with and without chronic kidney disease**

	Patients with CKD ( ***± s*** )	People without CKD ( ***± s*** )	***t***	***p-*** value
Age (years)	52.27 ± 13.18	43.78 ± 12.24	14.53	0.000
BMI (kg/m^2^)	25.78 ± 3.62	25.19 ± 4.01	4.68	0.000
SBP (mmHg)	137.01 ± 20.37	123.16 ± 16.52	14.12	0.000
DBP (mmHg)	83.19 ± 15.22	75.23 ± 11.85	8.19	0.000
Scr (μmol/L)	87.92 ± 56.72	77.43 ± 19.75	25.92	0.000
UA (μmol/L)	328.15 ± 88.62	308.19 ± 72.18	2.71	0.023
FBG (mmol/L)	5.43 ± 2.36	5.08 ± 1.30	13.16	0.000
TG (mmol/L)	2.03 ± 1.69	1.91 ± 1.43	5.01	0.000
TCH (mmol/L)	4.98 ± 1.23	4.75 ± 0.88	5.73	0.000
HDL-C (mmol/L)	1.27 ± 0.53	1.27 ± 0.52	0.22	0.762
LDL-C (mmol/L)	3.03 ± 0.94	2.73 ± 0.93	4.12	0.000
eGFR (mL/min/1.73 m^2^)	90.12 ± 28.19	110.32 ± 24.72	-27.17	0.000

BMI, body mass index; SBP, systolic blood pressure; DBP, diastolic blood pressure; Scr, serum creatinine; UA, uric acid; FBG, fasting blood glucose; TG, triglyceride; TCH, total cholesterol; HDL-C, density lipoprotein-cholesterol; LDL-C, low density lipoprotein-cholesterol; eGFR, estimated glomerular filtration rate.

Independent factors for CKD, as well as for hematuria, albuminuria and reduced eGFR, were analyzed by multivariate logistic regression analysis (Table 3). Factors independently associated with CKD included older age, female gender, smoking, high SBP and a history of diabetes mellitus, hyperlipidemia, hypercholesterolemia, hyperuricemia, and lower level of education. Older age, male gender, obesity, high SBP, hyperlipidemia, and smoking were independent risk factors for albuminuria; female gender and high SBP were risk factors for hematuria; and older age, female gender, and histories of cardiovascular disease, diabetes mellitus, hyperlipidemia, hypercholesterolemia, and hyperuricemia were independent risk factors for reduced eGFR.

**Table 3:** **Factors independently associated with albuminuria, haematuria, reduced eGFR, and chronic kidney disease among subjects in Taian**

	Albuminuria OR (95% CI)	Haematuria OR (95% CI)	Reduced eGFR ^a^ OR (95% CI)	CKD OR (95% CI)
Age	1.11 (1.03-1.36)	-	2.38 (2.13-2.63)	1.27 (1.16-1.40)
Female	0.61 (0.51­0.73)	3.12 (2.43-3.83)	1.70 (1.19-2.41)	1.40 (1.18-1.63)
Obesity	1.25 (1.12-1.41)	-	0.82 (0.73-0.94)	-
High SBP	2.23 (1.82-2.68)	1.52 (1.07-2.17)	-	1.72 (1.41-2.09)
Cardiovascular disease	-	-	2.5 (1.29-5.03)	-
Using nephrotoxic drugs	-	-	-	-
Diabetes mellitus	1.91 (1.53-2.31)	-	1.77 (1.36-2.18)	2.06 (1.53-2.62)
Hyperlipidemia	1.24 (1.03-1.45)	-	1.48 (1.02-2.03)	1.28 (1.05-1.63)
Hypercholesterolemia	-	-	1.59 (1.20-2.03)	1.18 (1.08-1.29)
Hyperuricemia	-	-	3.50 (2.12-4.89)	1.44 (1.09-1.86)
Smoking	1.38 (1.12-1.87)	-	-	1.29 (1.06-1.64)
≥ High school education	-	-	0.61 (0.39-0.95)	0.75 (0.65-0.87)

SBP, systolic blood pressure.

^a^mL/min/1.73 m^2^.

## Discussion

This is the first surveillance of CKD patients among subjects who visit selected hospitals in Taian City for primary care. This cross-sectional study evaluating the epidemiology of CKD and factors associated with it from September to December 2012. The key findings of this study were that 9.49% of the representative participants in Taian City had at least one indicator of CKD, whereas only 1.4% were aware that they had this disease. Factors associated with CKD, including diabetes mellitus, hypertension and educational level, were identified.

Our finding, that the prevalence of CKD in Taian City was 9.49%, was consistent with the first national survey in China, which showed that the overall prevalence of CKD was 10.8% [7]. However, studies in other provinces and municipalities in China have reported higher rates of CKD, 10.1% to 13.5% in Beijing, Zhejiang and Guangdong, and 19.1% in Tibet [18–20, 22]. Most of these regions are economically developed. The prevalence of diabetes mellitus has been found to increase substantially with economic development, from 5.8% in underdeveloped regions to 12.0% in developed regions [15]. A recent survey on the prevalence of CKD with type 2 diabetes (T2DM) in US adults found that eGFR was reduced or albuminuria present in 43.5% of the population, and that CKD was present in 61.0% of persons aged ≥ 65 years [25]. Our survey results indicated that diabetes mellitus was the strongest risk factor for CKD (OR = 2.06), which may explain the higher prevalence of CKD in Beijing, Zhejiang and Guangdong. Beijing is located in the north of China and its residents have a higher sodium intake in their diets [26, 27]. Sodium intake has been associated with increased blood pressure [28]. Our survey indicated that the second strongest risk factor for CKD was high SBP (OR = 1.72), an indicator of hypertension and commonly found in subjects with progressive CKD. A retrospective study of 540 Chinese patients with CKD found that 39.6% had hypertension [29]. Unmeasured confounders associated with specific geographical regions may also contribute to variations in the prevalence of CKD. For example, hypoxia is common in populations living at high altitude, such as in Tibet. Laboratory studies have shown that, with the development of hyperuricemia, exposure of rats to intermittent hypobaric hypoxia could lead to renal microvascular and tubulointerstitial injury, suggesting that hypoxia itself may cause low-grade renal injury [30].

Although the prevalence of CKD in Taian was high, only 1.4% of individuals with CKD were aware that they had this disease. This awareness rate was much lower than the rates reported in Thailand (1.9%) [17], India (7.9%) [31], and Saudi Arabia (7.1%) [32], and in a national survey throughout China (12.5%) [7]. Our survey was performed on a primary care population, with most of these patients with CKD being asymptomatic or having few clinical symptoms, which may help to explain the low disease awareness. Tests to detect early stage CKD are available, but have not yet been used widely, and most individuals do not undergo regular urine testing. Lack of healthcare resources must be considered. The World Health Organization (WHO) has reported that, in developing nations, government spending on health care was only 0.4% to 4% of the gross national product, far below the 10% to 16% in developed countries [33]. Compared with those unaware of having CKD, participants who were aware of their condition were more educated [34]. Our results also showed that higher educational level may be protective in CKD patients (OR = 0.75). Because less educated individuals have fewer health information contacts, they often pay less attention to self-care advice and are less aware of the occurrence of chronic disease, which may delay treatment. Long-term reduction of CKD morbidity and mortality requires more attention to early detection and prevention at the population level, especially in less educated subjects [35, 36].

This study used a multi-stage stratified cluster sampling procedure to obtain a representative sample of the primary care population in Taian City. All participating staff received intensive training before starting the survey and standardized protocols were used to ensure the quality of collected data. Additional advantages include the high response rate and the use of the MDRD equation modified for Chinese patients. All of these factors enhanced the credibility of the study results.

However, this study had several limitations. First, only one blood and one urine sample were obtained from each participant, making it difficult to determine whether hematuria or albuminuria was transient or persistent. Second, this study was cross-sectional and not longitudinal, preventing determination of whether any risk factors were the cause or result of CKD. Finally, this study was based on a primary care population; thus, the correct sampling weights were not used for insufficient data, thus limiting the generalization of our results to the general population of Taian.

## Conclusions

In conclusion, this is the first epidemiological survey of CKD in a large primary care population in Taian, China. The prevalence of CKD was found to be 9.49%, while the rate of awareness was much lower. Age, gender, educational level, smoking, SBP and history of diabetes mellitus, hyperlipidemia, hypercholesterolemia and hyperuricemia were independently associated with CKD. The prevalence of CKD may be reduced by controlling the increasing incidence of diabetes and hypertension in Taian. Health education and preventive policies for the general population are imperative. Rigorous designed studies with longitudinal data are required to confirm our results.

## Abbreviations

CKD: Chronic kidney disease
eGFR: estimated glomerular filtration rate
K/DOQI: Kidney Disease Outcomes Quality Initiative
NFK: National Kidney Foundation
ESRD: End-stage renal disease
SBP: Systolic blood pressure
DBP: Diastolic blood pressure
BMI: Body mass index
FBG: Fasting blood glucose
TCH: Total cholesterol
TG: Triglyceride
HDL-C: High density lipoprotein-cholesterol
LDL-C: Low density lipoprotein-cholesterol
UA: Uric acid
Scr: Serum creatinine
HLP: Hyperlipidemia
SD: Standard deviation
ORs: Odds ratios
CIs: Confidence intervals
T2DM: type 2 diabetes
WHO: World Health Organization.
